# Transitions among Health States Using 12 Measures of Successful Aging in Men and Women: Results from the Cardiovascular Health Study

**DOI:** 10.1155/2012/243263

**Published:** 2012-10-21

**Authors:** Stephen Thielke, Paula Diehr

**Affiliations:** ^1^Department of Psychiatry, University of Washington, Seattle, WA 98195, USA; ^2^Geriatrics Research, Education, and Clinical Center, Puget Sound VA Medical Center and Psychiatry and Behavioral Sciences, University of Washington, P.O. Box 356560, Seattle, WA 98195, USA; ^3^Departments of Biostastitics and Health Services, University of Washington, Seattle, WA 98195, USA

## Abstract

*Introduction*. Successful aging has many dimensions, which may manifest differently in men and women at different ages. *Methods*. We characterized one-year transitions among health states in 12 measures of successful aging among adults in the Cardiovascular Health Study. The measures included self-rated health, ADLs, IADLs, depression, cognition, timed walk, number of days spent in bed, number of blocks walked, extremity strength, recent hospitalizations, feelings about life as a whole, and life satisfaction. We dichotomized variables into “healthy” or “sick,” states, and estimated the prevalence of the healthy state and the probability of transitioning from one state to another, or dying, during yearly intervals. We compared men and women and three age groups (65–74, 75–84, and 85–94). *Findings*. Measures of successful aging showed similar results by gender. Most participants remained healthy even into advanced ages, although health declined for all measures. Recuperation, although less common with age, still occurred frequently. Men had a higher death rate than women regardless of health status, and were also more likely to remain in the healthy state. *Discussion.* The results suggest a qualitatively different experience of successful aging between men and women. Men did not simply “age faster” than women.

## 1. Introduction

Changes in health status, symptoms, and functioning during aging defy simple description. Despite the inevitability of death, no orchestrated or predictable decrements in health or types of sickness uniformly precede it, and individuals vary widely in how and how successfully they age. Certain groups may experience aging and health differently from others. The most obvious instance is gender: older men and women have been observed to have different lifespans [1, 2], functional trajectories [3], risk factors for disease [4], chronic and acute diseases [5, 6], and use of medical treatments [7]. There is practical and theoretical importance in understanding health changes during aging, especially the maintenance of health and the ability to recuperate from sickness, differ between men and women.

Successful aging is a multidimensional construct, which initially centered on absence of disease and objective physical and social functioning [8]. Recent research has deemphasized medical disease and concentrated more on self-identified successful aging, psychological health, well-being, mobility, and the absence of frailty [9–12]. There is no canonical definition for successful aging, and no single domain or variable captures all of its facets. Most previous analyses of age and gender effects on health status have examined health transitions for single health measures such as disability, depression, self-rated health, body mass index, ADL, or IADLs [13–21]. Because these studies generally focus on a single aspect of successful aging, and they inconsistently examine gender and age as predictors, they offer somewhat limited perspectives and generally cannot be compared.

There is utility in systematically scrutinizing the changes in different aspects of health status that occur with advancing age among men and women. First, evidence from research can help to counter ageist or sexist stereotypes about health during the aging process, which may influence social expectations, clinical care, and patient decision making [22]. Second, interventions to reduce morbidity and mortality can be better targeted by understanding the prevalence, persistence, and resolution of sickness in different populations. Third, observational results can inform both the theory of successful aging and research into the biology of aging. For instance, research has suggested that men “age faster” than women [23], yet this observation has not received much scrutiny in observational studies.

We sought to characterize a variety of measures of successful aging among a group of older adults, with particular attention to the differences between men and women of different ages. To explore changes in health systematically, and to allow age and gender comparisons, we analyzed transition probabilities for 12 different health variables which capture different aspects of psychological, physical, cognitive, and functional status. We hypothesized, for these 12 different domains of successful aging, that (1) the prevalence of health decreases because both the probability of remaining healthy and the probability of returning to a healthy state decline with increasing age, and (2) the prevalence and transition probabilities are different for men and women.

## 2. Methods

### 2.1. Data

Data came from the Cardiovascular Health Study (CHS), a population-based longitudinal study of risk factors for heart disease and stroke in 5888 adults aged 65 and older at baseline [24]. Participants were recruited from a random sample of Medicare eligible adults in four US communities, and extensive data were collected during annual clinic visits and telephone calls. The original cohort of 5201 participants, recruited in 1989 and 1990, had up to ten annual clinic examinations. A second cohort of 687 African Americans, enrolled in 1993 and 1994, had up to seven annual examinations.

The 12 variables used in this study were measures of health based on self-report or observation. They were selected in order to capture various aspects of psychological, physical, cognitive, and functional status. All were measured annually. Each value was dichotomized into “Healthy” and “Sick”, as shown in Table 1, using either previously published cutoffs or logical thresholds [27]. Some of the domains involved responses inherently suggestive of sickness. For hospitalization (HOSP), bed days (BED), ADL limitations (ADL), IADL limitations (IADL), and poor extremity strength (EXSTR), participants who endorsed the presence of any difficulty or one or more impairments were considered to be sick in that domain. For feelings that life is worthwhile (FLW), answers of “delighted,” “pleased,” and “mostly satisfied” were considered healthy; those of “mostly dissatisfied,” “unhappy,” and “terrible” were considered sick. For satisfaction with the purpose of life (SPL), using a 10-point scale between “extremely satisfied” (1) and “not at all satisfied” (10), answers better than “neither satisfied nor unsatisfied” (<4) were used to characterize health. For depression (DEP), a score of less than 10 on the CES-D depression scale was used to characterize health [20, 28]. For self-rated health (SRH), self-reported “excellent,” “very good,” or “good” general health were defined as healthy, and “fair” or “poor” as sick [29]. Healthy cognitive status (COG) was defined as above 89 points on a 100-point scale, corresponding to 26 or higher on the MMSE, which is suggestive of intact cognition [30]. The sick state for ambulation (TWLK) was defined by a velocity of less than 0.66 feet per second (10 seconds) in a 15-foot walk, which is a marker for low speed [31]. Blocks walked during the last week (BLK) were dichotomized at 28 blocks or more to signify the healthy state [32]. Because the analyses compared age and gender groups within each domain rather than the domains to each other, the variability in the prevalence of healthy and sick states as a result of different cutoffs were unlikely to influence the main results significantly.

In order to simplify comparisons, age was divided into three categories—65–74, 75–84, and 85–94—in accordance with the common definitions of “young old,” “old old,” and “oldest old”. Persons could contribute data to more than one age category, depending on their age at the start of each transition.

Missing data were imputed, after a transformation to recode death as zero health, by interpolating over time between existing data points for each person. Any data that remained missing at the end of a sequence were extrapolated as an average of the last observed value and of transformed self-rated health (which was measured every 6 months and is thus well characterized) [33, 34]. No imputation across participants was performed. All available observations were used, including those that were imputed.

### 2.2. Statistical Approach

**Figure 1:**
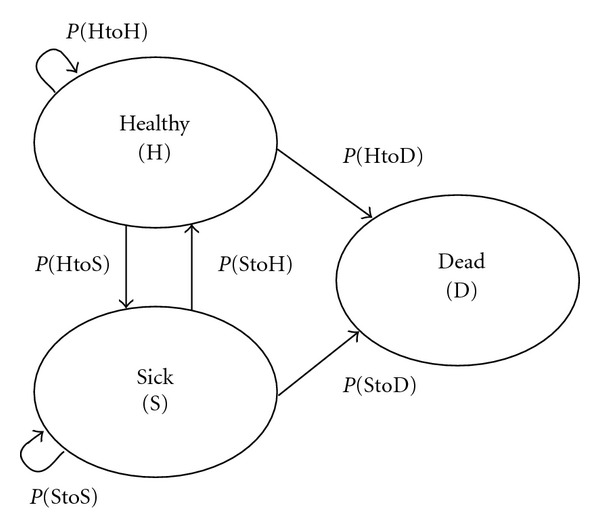
Transition probability model, showing the probability of remaining in a state or moving to another state during specific time intervals (such as one year).

In order to represent and compare prevalence and incidence of healthy and sick states in various domains, and to account for death, we used a transition probability approach. This technique has been used to examine other changes in health among older adults, including self-rated health [13], obesity [14], depression [20], and pain [35]. Transition probability models make the simple assumption that health status measures can be categorized into discrete states, among which individuals can move. The ovals in Figure 1 represent discrete health states, and the arrows show the likelihood of transitioning from one state to another during a single time interval, one year. The number of individuals in each of the states (shown by the ovals) is the relative prevalence of health or sickness in the total population.

When evaluated at two time points, there are thus six possible transitions among these states: remaining healthy (P(HtoH)), becoming sick (P(HtoS)), remaining sick (P(StoS)), becoming healthy (P(StoH)), dying from a state of health (P(HtoD)), and dying from a state of sickness (P(StoD)). Persons move among those states with certain probabilities, which may vary by age, gender, or other characteristics. The equilibrium—or steady state—prevalence of a system can be calculated directly from the transition probabilities [36].

### 2.3. Analysis

The prevalence of the healthy state of each variable in each wave was calculated as the percentage of living persons who were healthy. The one-year probabilities of transitioning from state to state were estimated from crosstabulation of data collected one-year apart. All cases where a beginning state and a starting state were available were used, for all 5888 participants. General patterns by age and gender groups were described. We estimated one-year transition probabilities for participants starting in each health state (sick or healthy). These transition probabilities, shown in Figure 1, were constructed as a simple fraction: number of moving to other state or remaining in state/number in the state at the starting time point. Calculations were performed separately for each age and gender grouping. The associations of the estimated transition probabilities with age and gender were tested using cross-sectional time series logistic regression (the xtlogit command in Stata). This form of generalized estimating equation accounts for participants contributing multiple observations. We carried out separate analyses for men and women of the same age group and estimated the statistical significance of the difference using generalized estimating equations.

The level of significance for individual comparisons was set at *P* < 0.05, two tailed, and differences between men and women in the same age group at this degree are shown in bold in the tables. The differences by age are described in the text. We summarize the significant findings across variables, with the reminder that 5% of all significant differences could be due to chance. There was no explicit adjustment for multiple comparisons, because all of the results were shown (i.e., we did not select only the significant results after running multiple models and because of the large number of potential combined hypotheses.) 

Analyses were conducted in Stata (StataCorp, College Station, Texas, version 11.2).

## 3. Findings

45,297 transition pairs (starting and ending state, separated by one year) from the 5888 participants were analyzed. During the study, 1684 participants died. For the whole sample, 13.5% of observations were missing and imputed, with some variability across the 12 domains of health. In the year prior to death, 7% of all observations were missing, which were part of the imputed fraction. The median number of imputed observations per participant was one. 

**Figure 2:**
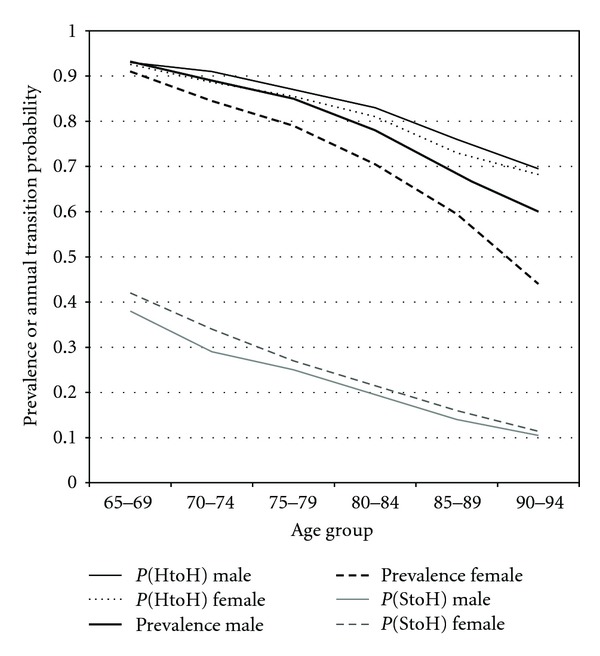
Prevalence, maintenance, and recovery for ADL Health by age and sex. The prevalence estimates are the proportion with no ADL difficulties. The transition probability estimates are the likelihoods of remaining healthy (P(HtoH)) and of returning to health (P(HtoS)) over one-year intervals.

As an illustration of the analytic approach, the prevalences and selected transition probabilities for ADL are shown in Figure 2. “Healthy” was defined as having no difficulties with activities of daily living, and “sick” as having one or more difficulties. The third and fourth lines from the top represent the healthy prevalence (proportion of the living who had no ADL difficulties), with a solid line for males and a dotted line for females. The prevalence is quite high for the youngest group (about 90%) and declines over time. The healthy prevalence is higher for men than for women, and the difference becomes larger with age. The lowermost two lines represent the probability of recovering from the sick state (having ADL difficulties) by transitioning into the healthy state (no ADL difficulties) one year later, labeled as P(StoH). The probability is initially near 0.4 and is higher for women than for men. The two topmost lines in the graph represent the probability of staying in the healthy state, P(HtoH). This probability is initially about 0.9 and is higher for men than for women. This figure shows how transitions and prevalence both change over time and differ between men and women, and also how relatively small differences in transitions accumulate into more pronounced differences in prevalence.

### 3.1. Prevalence of the Healthy State

The first two lines of Table 2 show the number of observations (transition pairs) in each group, and the mean age, by age category and gender. Mean age did not differ significantly in each category for men and women. The next 12 lines show the prevalence of a healthy state for each variable. For example, for HOSP, 91.3% of the women aged 65–74 were “healthy,” defined as “having no hospital days.” For men in the same age range, the prevalence of a healthy state was 88.0%. Over the three age groups, women's prevalence for HOSP declined from 91.3% to 87.8% to 84.9%.

Prevalence by Age and Gender. All of the prevalence values in Table 2 declined with age; the prevalence values were significantly lower at each subsequent age group compared with the younger one. The bolded entries in Table 2 show the situations where women or men had a significantly higher prevalence of a healthy state. Women were significantly healthier than men only for HOSP and COG. Men had a significantly higher prevalence of a healthy state than women for all the 33 other domains and age groups, except for three where there was no significant difference. 

### 3.2. Transitions Probabilities for Healthy Persons

Table 3 shows the transition probabilities for persons who were initially in the healthy state. For instance, for HOSP, age 65–74, women who were healthy (without a hospital stay in the first year) had a 0.92 probability of remaining healthy (not having a hospital stay in the next year), a 0.08 probability of becoming sick (having a hospital stay), and a 0.01 probability of dying. The bold entries in the upper and lower tables represent probabilities where women or men became or remained significantly healthier. More than half of the comparisons were found to be statistically significant.

In all three comparisons (remaining healthy, becoming sick, and dying from a healthy state), there was a significant decline with advancing age for almost all the domains of health. Men were more likely than women to remain healthy and less likely to become sick in the majority of comparisons, and men were more likely to die.

### 3.3. Transition Probabilities for Sick Persons

Table 4 shows the transition probabilities for persons who were initially in the sick state. The bolded entries indicate probabilities of remaining in or recovery from sickness, or probability of death, that were significantly healthier in women (in the top of the table) or in men (in the bottom). For convenience we considered P(StoS), remaining sick, as an unfavorable transition, because the person did not recover, but it could also be considered as favorable because the person did not die.

In almost all domains of health, the probability of recovery from a sick state declined significantly with age, while the probability of dying from a sick state increased with age. Women were significantly more likely than men to recover from a sick state in six of 36 total groups; men were more likely to recover in 12. Men were less likely to remain in a sick state than women in 23 of the 36 groups; in no cases were women significantly less likely than men to remain sick. For every health variable, men were significantly more likely to die from a state of sickness than women were.

## 4. Discussion

### 4.1. Overview

This analysis, unlike previous approaches that have focused on one or a few domains of health, examined the prevalence of and transitions in 12 measures of successful aging among a cohort of older adults. The 12 health-related variables, while related, were diverse and included measures of physical and mental health, quality of life, and health behaviors. Some were self-reported and some were measured through objective tests. Some were subjective single item questions while others were based on structured responses. Despite these differences, most of the measures of successful aging performed in very similar ways with regard to their associations with age and gender.

### 4.2. Age Trends

Overall, the trends in prevalence suggest both high initial rates of health and consistent incremental declines in health with advancing age. First, the prevalence of emotional and functional health shown in Table 2 remained quite high, even at advanced ages. For instance, about 85% of 85–94-year-old individuals expressed the belief that life is worthwhile. Second, health declined with advancing age across all the domains: there were no areas that were spared the effects of aging. Some aspects declined more dramatically, especially the functional measures of cognition, timed walk, and blocks walked. In these domains, the proportion of participants who were healthy in the oldest group was less than half the proportion of who were healthy in the youngest group. Nevertheless, the majority of men and women aged 85–94 in this community cohort had no hospital days, no bed days, no ADL difficulties, were not depressed, had high self-rated health, and felt that life was worthwhile. These findings are a testament to overall successful aging among the old old and oldest old, and challenge stereotypes about the high prevalence of sickness and disability in these groups.

### 4.3. Prevalence and Incidence by Gender and Age

Men were observed to have a higher prevalence of a healthy state except for hospitalization and cognitive status. In Table 4, P(HtoS) is consistently higher for men than for women only for these domains of health; Table 3 shows smaller and less consistent differences in P(StoH). Most of the gender difference is thus driven by difference in incidence of sickness rather than difference in recovery from it. In other words, men seem to be healthier because they are less likely to transition *into* sick states, while men and women show roughly the same return to health. This implies an aging process for men in which the sickest persons are consistently removed by recovery or death, while for women the sickest persons are less likely to be removed. A different trend is seen for the age effects: with increasing age, the probability of staying healthy becomes lower than the probability of staying sick, which decreases the prevalence of health. Older adults are thus less healthy than younger adults mainly as a result of less likely recovery from sickness combined with higher probability of death. These findings may have some implications for disease prevention and treatment programs [37]. 

### 4.4. Patterns of Aging in Men and Women

 We found several key differences in aging based on gender. Men died more often than women from a state of either sickness or health, remained more healthy than women while alive, and were more likely to recover from being sick. These differences were not characterized by similar transitions offset by a period of years, as might happen if men's health trajectories were simply premature or accelerated versions of women'strajectories. Comparing the adjacent age categories for men and women (for instance, 65–7-year-old men compared to 75–84-year-old women) shows that younger men's transitions were more similar to older women'stransitions than younger women's transitions to older men's transitions for 58 (60%) of the 96 possible comparisons. This is seen graphically for ADL status in Figure 2, where the transition probabilities for men are consistently higher for P(HtoH) but lower for P(StoH), and there is no transformation of the women's transitions (as by moving the lines for women to the left, or by compressing the curves) that would make them match those for men.

These results demonstrate qualitative differences in health transitions between men and women during aging, not just that men “age faster”, as has been suggested [23]. If this had been the case, men would have developed sickness sooner than women, remained sick as much as women, and had equal likelihood of dying from a state of sickness as women. Not only was this effect not seen but also in many cases opposite effects were observed. The findings suggest that men may show more compression or squaring of mortality than women [38]. Put another way, the occurrence of sickness—regardless of the operational definition—does not seem to be the key intermediary which makes men die sooner than women, and no other factor associated with gender well accounts for this effect.

The observed differences in incidence and prevalence of health between men and women encourage speculation about their etiology in human gender dimorphism and argue for fundamental differences between how men and women age. Historical analyses suggest that the environmental pressures of infectious disease and resource availability have caused women to live longer [39]. Some research suggests that lower mortality among women could be attributable to a low-risk, healthy lifestyle [40, 41], or to lack of male hormones such as testosterone [42]. Other factors include the unequal distribution of chronic conditions, health behaviors like smoking, and differential effects of diseases on mortality [40]. Women may assess their health differently, for instance by applying different anchoring points to define sick and healthy states, or by being more willing to assume the sick role. [43, 44]. This latter possibility is supported by the fact that hospitalization and cognitive status, the only variables where women were healthier than men, are relatively objective measures. Nonetheless, timed walk, another objective measure of health, favored men. In order to understand the underlying factors behind these gender differences better, it might be useful to repeat these analyses using other variables, datasets, and age groups, with attention to differences in self-reported and performance measures.

### 4.5. Limitations

First, about one-tenth of the data were missing and had to be imputed. The ascertainment of death, however, was essentially complete. The approach we used for imputation, using interpolation and extrapolation, may have minimized changes by assuming that the missing health status was mainly consistent with the data before and after the missing measurement and declined close to death. There was no difference in missingness between men and women, and it is unlikely that this imputation method would intensify group differences. Second, the individual significance tests that are reported here should be considered as descriptive rather than definitive, due to the large number of comparisons that were made in this analysis. Third, the category of “healthy” in the various outcome measures should not be interpreted literally, since the cutoffs for healthy and sick states were assigned by categorizing each variable individually, and not by cross-validating them with other metrics for subjective or objective health. Most of the cutoffs have face validity as markers of health. Fourth, in the interests of generating straightforward descriptive results, we did not adjust for other patient-level health or sociodemographic characteristics which might confound the associations between age, gender, and health. Investigating these could help understand how men and women differ in the aging process. 

## 5. Summary and Conclusion

Transition probabilities among various states of health during aging, and separately for men and women, are rarely reported in ways that can be compared. We calculated the gender-specific transition probabilities for 12 health-related variables across different domains of health and believe that these have utility for organizing future research and for characterizing the changes that happen during aging. In general, the 12 variables all behaved similarly. Recuperation from sickness declined with age, but still occurred frequently. Men and women experienced different types of change in health over time, with men showing more health and less sickness, but greater likelihood of dying. Men did not simply age faster compared to women. They exhibited a more “square” pattern of health status over time, with dropoff at younger ages than women. There is no simple explanation for the differences observed between men and women; they may partly relate to women's health-related behaviors or perceptions of health, or to more biologically fundamental gender dimorphisms. Future study can help to clarify how health is constructed and changes in different groups during aging.

## Figures and Tables

**Table 1:** 12 Measures of successful aging.

Category	Abbreviation	Question	Definition of healthy
Not hospitalized	HOSP	“Did you stay overnight in the hospital in the last 6 months?”	No report of being hospitalized
No bed days	BED	“During the past two weeks, how many days have you stayed in bed all or most of the day because of illness or injury?”	No days in bed reported
Life satisfaction	SPL	“How satisfied are you with the meaning and purpose of your life? on a scale of 1–10, with 1 being extremely and 10 being not at all”	Score of 1 to 4
Life as a whole	FLW	“How do you feel about life as a whole?” (1: delighted; 3: mostly satisfied; 6: terrible)	Score of 1–3
Not depressed	DEP	10 questions of the center for epidemiologic studies short depression scale [25], each ranked 0–3	Score < 10, out of a possible 30 points
No limitations in activities of daily living	ADL	“Do you have any difficulty performing this activity?” from a list of walking, transferring, eating, dressing, bathing, or toileting	No difficulties reported
No limitations in independent activities of daily living	IADL	“Do you have any difficulty performing this activity?” from a list of heavy or light housework, shopping, meal preparation, money management, or telephoning	No difficulties reported
Intact extremity strength	EXSTR	“Do you have any difficulty with this activity” from a list of lifting, reaching, or gripping	No difficulty reported
Self-rated health	SRH	“How would you rate your health in general: excellent, very good, good, fair, or poor?”	Excellent, very good, or good self-reported health
Intact cognition	COG	Modified minimental state examination [26], scored from 0 to 100	Score above 89
Ability to ambulate	TWLK	Timed 15 foot walk	Less than 10 seconds
Frequent ambulation	BLK	“During the last week, how many city blocks did you walk?”	>4 blocks per day, on average

**Table 2:** Prevalence of a healthy state among men and women, with health defined separately for each domain. Bolded entries represent significantly higher prevalence of health in women (left columns) or men (right columns). The differences between groups based on age are described in the text.

	Female			Male		
	65–74	75–84	85–94	65–74	75–84	85–94
Number of observations	12261	12433	2183	7801	8867	1752
Mean age	71.0	78.6	87.5	71.1	78.6	87.7
HOSP: no hospital days	**91.3**	**87.8**	**84.9**	88.0	85.6	81.7
BED: no bed days	94.3	92.3	89.3	**95.9**	**94.6**	**91.3**
SPL: Satisfied with purpose of life	75.7	69.8	59.6	**80.7**	**75.2**	**67.4**
DEP: not depressed	80.2	73.8	64.3	**87.6**	**81.4**	**70.9**
ADL: no ADL difficulties	86.3	76.1	56.7	**90.2**	**82.7**	**67.6**
FLW: feel life is worthwhile	94.4	91.0	83.6	**95.3**	91.7	85.6
EXSTR: good extremity strength	67.6	57.6	42.1	**84.6**	**78.8**	**65.4**
SRH: high self-rated health	79.0	70.8	60.7	80.0	**73.6**	**64.7**
TWLK: walk 10 feet < 10 seconds	64.3	44.5	16.9	**73.8**	**58.6**	**30.0**
IADL: no IADL difficulties	71.8	58.7	39.5	**80.9**	**70.5**	**50.7**
COG: 3 MSE > 90	**70.9**	**54.6**	**27.6**	67.2	53.1	25.7
BLK: walked 4+ blocks per day	33.3	20.9	8.5	**47.7**	**37.8**	**22.3**

**Table 3:** One-year transition probabilities for those starting in a healthy state for 12 different variables. Bolded entries indicate a significantly healthier transition (more likely remaining healthy, less likely remaining sick, or less likely dying) among women compared to men (top half) or men compared to women (bottom half). The differences between groups based on age are described in the text.

	Age 65–74			Age 75–84			Age 85–94		
	P(HtoH)	P(HtoS)	P(HtoD)	P(HtoH)	P(HtoS)	P(HtoD)	P(HtoH)	P(HtoS)	P(HtoD)
Female									

HOSP	**0.92**	**0.08**	**0.01**	**0.88**	**0.10**	**0.02**	**0.80**	**0.13**	**0.07**
BED	0.95	0.05	**0.01**	0.92	0.06	**0.02**	0.86	0.08	**0.06**
SPL	0.85	0.15	**0.01**	0.80	0.18	**0.02**	0.70	0.27	**0.04**
DEP	0.88	0.11	**0.01**	0.84	0.14	**0.02**	0.77	0.19	**0.04**
ADL	0.90	0.09	**0.01**	0.85	0.14	**0.02**	0.72	0.24	**0.04**
FLW	**0.96**	0.04	**0.01**	0.92	0.06	**0.02**	**0.85**	0.11	**0.05**
EXSTR	0.83	0.17	**0.01**	0.77	0.21	**0.02**	0.66	0.30	**0.03**
SRH	**0.90**	**0.30**	**0.01**	0.85	0.37	**0.01**	0.73	**0.40**	**0.04**
TWLK	0.83	0.17	**0.00**	0.72	0.27	**0.01**	0.54	0.45	**0.01**
IADL	0.85	0.15	**0.01**	0.77	0.21	**0.02**	0.61	0.35	0.05
COG	**0.86**	**0.13**	**0.01**	**0.81**	**0.18**	**0.01**	0.67	0.30	0.04
BLK	0.65	0.01	**0.00**	0.55	**0.01**	**0.01**	0.49	0.03	0.03

Male									

HOSP	0.88	0.10	0.02	0.85	0.12	0.03	0.76	0.15	0.09
BED	0.95	**0.03**	0.02	0.92	**0.04**	0.04	0.84	0.07	0.10
SPL	**0.86**	**0.12**	0.02	**0.82**	**0.16**	0.03	0.71	**0.21**	0.08
DEP	**0.91**	**0.08**	0.02	**0.86**	**0.11**	0.03	0.76	**0.16**	0.07
ADL	**0.92**	**0.06**	0.02	**0.87**	**0.11**	0.03	0.75	**0.18**	0.07
FLW	0.95	**0.03**	0.02	0.92	0.05	0.03	0.82	0.10	0.08
EXSTR	**0.90**	**0.08**	0.02	**0.85**	**0.12**	0.03	0.74	**0.20**	0.06
SRH	0.89	0.35	0.01	0.84	**0.34**	0.03	0.75	0.45	0.06
TWLK	**0.85**	**0.13**	0.02	**0.76**	**0.22**	0.02	0.61	**0.34**	0.05
IADL	**0.88**	**0.11**	0.01	**0.81**	**0.17**	0.02	0.64	**0.30**	0.06
COG	0.83	0.15	0.02	0.78	0.20	0.03	0.62	0.33	0.05
BLK	**0.72**	0.02	0.01	**0.63**	0.02	0.02	0.54	0.04	0.03

**Table 4:** One-year transition probabilities for those starting in a sick state for 12 different variables. Bolded entries indicate a significantly healthier transition (more likely becoming healthy, less likely remaining sick, or less likely dying) among women compared to men (top half) or men compared to women (bottom half). The differences between groups based on age are described in the text.

	Age 65–74			Age 75–84			Age 85–94		
	P(StoH)	P(StoS)	P(StoD)	P(StoH)	P(StoS)	P(StoD)	P(StoH)	P(StoS)	P(StoD)
Female									

HOSP	0.68	0.26	0.06	**0.61**	0.29	**0.11**	0.54	0.27	**0.20**
BED	0.55	0.37	**0.08**	**0.44**	0.41	**0.15**	0.34	0.37	**0.30**
SPL	0.37	0.60	**0.03**	0.30	0.64	**0.06**	0.24	0.61	**0.16**
DEP	0.35	0.62	**0.03**	0.29	0.64	**0.07**	0.20	0.63	**0.17**
ADL	**0.36**	0.59	**0.05**	0.25	0.67	**0.08**	0.15	0.70	**0.15**
FLW	**0.37**	0.54	**0.09**	**0.29**	0.56	**0.15**	0.18	0.53	**0.29**
EXSTR	0.31	0.66	**0.03**	0.23	0.72	**0.05**	0.14	0.74	**0.12**
SRH	0.31	0.64	**0.04**	0.25	0.67	**0.07**	**0.24**	0.61	**0.15**
TWLK	0.29	0.68	**0.03**	0.16	0.80	**0.05**	0.06	0.84	**0.10**
IADL	0.30	0.67	**0.03**	0.22	0.73	**0.05**	0.16	0.73	**0.11**
COG	0.27	0.70	**0.03**	0.17	0.78	**0.05**	0.07	0.83	**0.11**
BLK	0.14	0.84	**0.02**	0.08	0.88	**0.04**	0.03	0.88	**0.09**

Male									

HOSP	0.63	0.29	0.08	0.56	0.28	0.16	0.49	0.23	0.28
BED	0.49	0.35	0.15	0.33	0.36	0.32	0.26	0.33	0.41
SPL	**0.41**	**0.53**	0.06	0.32	**0.56**	0.12	0.24	**0.54**	0.22
DEP	0.37	**0.54**	0.08	0.27	**0.57**	0.16	0.18	0.58	0.25
ADL	0.31	0.59	0.10	0.23	**0.60**	0.17	0.13	**0.63**	0.24
FLW	0.29	0.55	0.16	0.20	0.52	0.28	0.15	0.46	0.39
EXSTR	**0.37**	**0.57**	0.07	**0.31**	**0.56**	0.14	**0.20**	**0.56**	0.24
SRH	0.32	**0.60**	0.08	0.25	**0.62**	0.13	0.18	0.59	0.24
TWLK	**0.34**	**0.61**	0.05	**0.21**	**0.70**	0.09	**0.08**	**0.76**	0.16
IADL	**0.34**	**0.59**	0.08	**0.24**	**0.64**	0.12	0.17	**0.64**	0.19
COG	0.27	0.69	0.04	0.18	**0.74**	0.08	0.07	**0.78**	0.15
BLK	**0.21**	**0.76**	0.04	**0.14**	**0.79**	0.07	**0.08**	**0.77**	0.15
